# The link between chronic cocaine use, B cell perturbations, and blunted immune recovery in HIV-infected individuals on suppressive ART

**DOI:** 10.1515/nipt-2022-0019

**Published:** 2023-03-25

**Authors:** Da Cheng, Zhenwu Luo, Sylvia Fitting, William Stoops, Sonya L. Heath, Lishomwa C. Ndhlovu, Wei Jiang

**Affiliations:** Department of Microbiology and Immunology, Medical University of South Carolina, Charleston, SC, USA; Department of Psychology & Neuroscience, University of North Carolina at Chapel Hill, Chapel Hill, NC, USA; Department of Behavioral Science, Department of Psychiatry, Center on Drug and Alcohol Research, Department of Psychology, University of Kentucky College of Medicine and College of Arts and Sciences, Lexington, KY, USA; Department of Medicine, Division of Infectious Diseases, University of Alabama at Birmingham, Birmingham, AL, USA; Department of Medicine, Division of Infectious Diseases, Weill Cornell Medicine, New York, NY, USA; Ralph H. Johnson VA Medical Center, Charleston, SC, USA; Divison of Infectious Diseases, Department of Medicine, Medical University of South Carolina, Charleston, USA

**Keywords:** anti-CD4 IgG, antibody-dependent cytotoxicity, antiretroviral therapy, CD4+ T cell recovery, cocaine, HIV

## Abstract

**Background:**

We recently reveal that anti-CD4 autoantibodies contribute to blunted CD4+ T cell reconstitution in HIV+ individuals on antiretroviral therapy (ART). Cocaine use is common among HIV+ individuals and is associated with accelerated disease progression. However, the mechanisms underlying cocaine-induced immune perturbations remain obscure.

**Methods:**

We evaluated plasma levels of anti-CD4 IgG and markers of microbial translocation, as well as B-cell gene expression profiles and activation in HIV+ chronic cocaine users and non-users on suppressive ART, as well as uninfected controls. Plasma purified anti-CD4 IgGs were assessed for antibody-dependent cytotoxicity (ADCC).

**Results:**

HIV+ cocaine users had increased plasma levels of anti-CD4 IgGs, lipopolysaccharide (LPS), and soluble CD14 (sCD14) versus non-users. An inverse correlation was observed in cocaine users, but not non-drug users. Anti-CD4 IgGs from HIV+ cocaine users mediated CD4+ T cell death through ADCC *in vitro*. B cells from HIV+ cocaine users exhibited activation signaling pathways and activation (cycling and TLR4 expression) related to microbial translocation versus non-users.

**Conclusions:**

This study improves our understanding of cocaine associated B cell perturbations and immune failure and the new appreciation for autoreactive B cells as novel therapeutic targets.

## Introduction

A subgroup of virologically suppressed HIV+ individuals fail to restore their CD4+ T cell counts to levels observed in demographically matched HIV uninfected individuals even under long-term antiretroviral therapy (ART). These individuals experience increased chronic inflammation, immune activation, and heightened mortality and morbidity risks. Numerous studies have investigated the mechanisms of poor CD4+ T cell recovery, including but not limited to fibrosis of thymic and lymphoid organs, persistent inflammation and immune activation, and virus-mediated effects [1, 2]. We were the first group to demonstrate the role of pathogenic anti-CD4 autoantibodies in ART-treated HIV as an associated mechanism of poor CD4+ T cell reconstitution [3].

Illicit drug use is common among HIV-infected individuals [4]. Cocaine affects HIV progression independent of treatment [5]. In HIV, cocaine use accelerates central nervous system, dysfunction of mitochondria and DNA methylation [6, 7], blood-brain barrier impairment [8], neurocognitive dysfunction, and neurodegeneration [9], [10], [11]. Individuals with cocaine use disorder show elevated proinflammatory cytokines both at baseline and following exposure to the stress imagery condition [12, 13]. Moreover, cocaine abuse is associated with enhanced HIV transcription and replication [14, 15], liver and heart organ damage, and distinct inflammatory profiles [16, 17], compared to HIV-infected individuals without history of substance use disorder. Notably, substance use disorder is associated with CD4+ T cell decline, likely stemming from loss of adherence to ART and uncontrolled viremia.

Toll-like receptor (TLR) signals in B cells play roles in pathogenic humoral autoimmunity [18]. Human B cells in general express high levels of toll-like receptor (TLR)7 and TLR9 but low levels of TLR2 and TLR4 [19]. TLR2, 4, 7, and 9 are involved in autoantibody production in autoimmune diseases [18, 20], [21], [22]. Altered B-cell receptor (BCR) and TLR signals (e.g., MyD88) may promote autoreactive B cell selection [23]. Moreover, increased microbial translocation and accelerated disease progression were found in HIV-infected subjects or animals with opioid use disorders compared to HIV+ non-opioid users or animals, suggesting that drug users may experience a more severe disruption of gut barrier in HIV infection [24]. However, the link between B cell perturbations, cocaine use, and ART outcomes in HIV remain unknown.

In the present study, we investigated the role of chronic cocaine use on B cell function, anti-CD4 autoantibody production in relation to poor CD4+ T cell recovery in HIV-infected individuals on viral-suppressive ART.

## Materials and methods

### Study subjects

The Institutional Review Board (IRB) was approved for the current study at the Medical University of South Carolina and University of Alabama at Birmingham. Consent was obtained from all participants. HIV-infected individuals were from Charleston, SC and Birmingham, AL; all were aged 18–60 years and on virologically suppressed ART, defined by plasma HIV RNA levels below detection limits, for at least one year. As shown in our previous studies [25], chronic cocaine use was identified through questionaries, self-reporting, and cocaine urine screens for cocaine positive only, performed using the onTrak test cup (cannabis, cocaine, amphetamines, opiates, and phencyclidine, TestCountry). The routes of cocaine use were either smoke inhalation or intranasal administration, but not through intravenous (IV) administration. The HIV+ non-drug use controls were identified by self-reporting and urine tests negative for all 5 drugs; they were matched by age and gender. We also test CD4+ T cell counts and anti-CD4 IgG in plasma samples from uninfected cocaine users and non-drug use controls from a previous study [25].

### Flow cytometry

Peripheral blood mononuclear cells (PBMC) were isolated from EDTA-containing blood using a Ficoll-Hypaque cushion (GE, Pittsburgh, PA). B cell cycling was assessed by surface staining with anti-CD19 antibody and then intracellular staining with ki67 or isotype control antibody (BD Biosciences) after membrane permeabilization (Fixation*/*Permeabilization Solution Kit, BD Pharmingen, San Jose, CA). Cells were collected by BD FACSVerse Flow Cytometer and analyzed by FlowJo software (Version 10.0.8).

### Gene expression profile analysis of human B cells

Human total B cells were isolated from PBMCs of 4 HIV+ non-users and 3 HIV+ cocaine users using B cell negative isolation kit (Miltenyi Biotec, San Diego, CA, purity >95%). Total RNA was extracted using RNeasy Plus Mini Kit (Qiagen, Germantown, MD) according to the manufacturer’s instructions. RNA qualitative and quantitative were analyzed using Qubit 3.0 (Life Technologies, Carlsbad, CA) and Tape station 4200 (Agilent Technologies, Santa Clara, CA). RNA concentration more than 20 ng/μL was selected for the Affymetrix GeneChip assays (Affymetrix, Santa Clara, CA). Briefly, Affymetrix Human GeneChip U133 Plus 2.0 Array was used for RNA hybridization and labeling assay according to the manufacturer’s instructions. The analysis of scanned images and signal values for each probe set were obtained using GCOS (Affymetrix). The microarray data analysis was performed in R program (Version 3.3.1). Limma package [26] was used to identify differentially expressed genes between HIV+ cocaine users and HIV+ non-users. Pathway enrichment was analyzed using Cytoscape software platform [26].

### Plasma levels of anti-CD4 IgG, LPS, and soluble CD14

The method of plasma anti-CD4 IgG measurement was published in our previous study [4]. Briefly, we coated the ELISA plate with human soluble CD4 protein (sCD4, Progenics, Tarrytown, NY). The ratio of 1:40 dilution for plasma, 1:5000 dilution for biotin labeled goat anti-human IgG, and 1:1000 dilution for horseradish peroxidase conjugated streptavidin (HRP-Streptavidin) were applied. sCD14 in plasma was evaluated by ELISA after 1:40 dilution (R & D system). LPS was quantified using a commercially available limulus amebocyte assay kit (Lonza Inc., Allendale, NJ) according to the manufacturer’s protocol.

### TLR4 and TLR9 mRNA expression in B cells

B cells were isolated using a negative selection kit (Miltenyi Biotec) and purity was above 96%. Total RNA from B cells was extracted using RNeasy Micro kit following the manufacturer’s protocol (Qiagen). TLR4 and TLR9 mRNA relative expression to GAPDH was assessed by qPCR. The primers of each target were shown in a previous study [19].

### Antibody affinity purification and antibody-dependent cytotoxicity (ADCC)

The details of antibody purification from plasma and ADCC assay were described in our previous study [4]. Briefly, NHS Mag Sepharose (GE Healthcare, Wauwatosa, WI) covalently coupled with sCD4 protein (Progenics) were used for high affinity anti-CD4 specific IgG purification, and protein A/G agarose beads were used for total IgG purification (Pierce, Pittsburgh, PA). The monoclonal anti-CD4 antibody Zanolimumab (HuMax-CD4, Genmab) was used to generate standard curves and to use as a positive control. Total IgGs from HIV+ non-drug users were used as negative controls. For ADCC, CD4+ T cells and NK cells were isolated using MACS (STEMCELL, Vancouver, Canada) and co-cultured at a ratio of 1:3 with purified anti-CD4 IgGs and control IgGs. CD4+ T cell apoptosis and cytolysis was evaluated by flow cytometry.

### Statistical analysis

Prism software (GraphPad 6.0, La Jolla, CA) was used for data analysis. The Mann–Whitney test (non-parametric) was applied for the comparisons between the two study groups [27]. Spearman correlation tests were applied for associations between pairs of continuous variables. All tests were 2-sided, and p<0.05 was considered to denote statistical significance.

## Results

### Anti-CD4 IgGs from HIV-infected cocaine users play a role in poor CD4+ T cell recovery following suppressive ART

**Figure 1:**
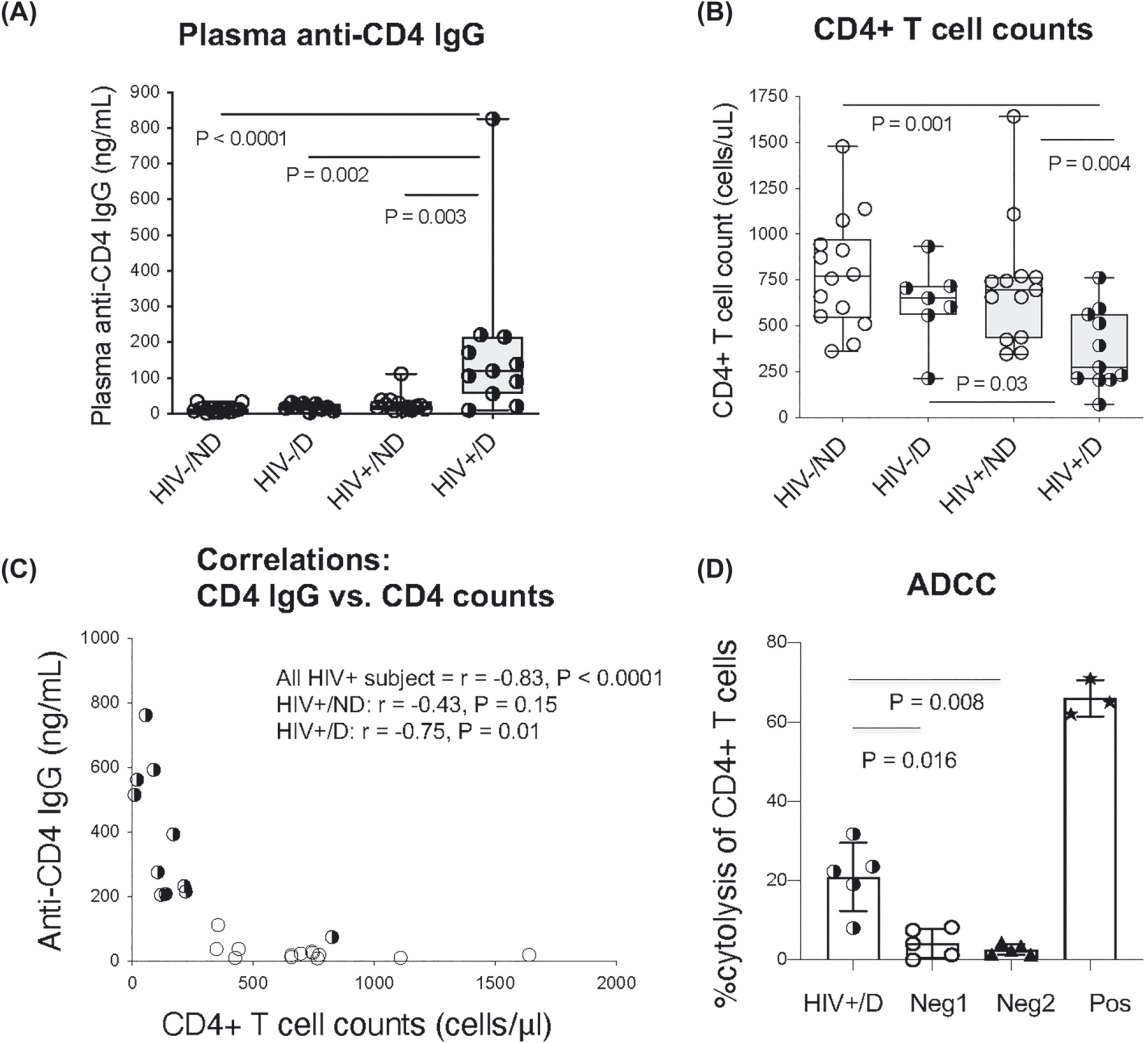
Increased plasma levels of anti-CD4 IgG in HIV+ cocaine users and its effect on CD4+ T cell death through ADCC. This study included HIV-negative cocaine users (HIV–/D) and non-users (HIV–/ND), and aviremic ART-treated HIV+ cocaine users (HIV+/D) and non-users (HIV+/ND). The median plasma anti-CD4 IgG levels (A) and CD4+ T cell counts (B) and their correlations in HIV+ subjects (C) were evaluated. (D) The ADCC was performed in CD4+ T and NK cells in the presence of 5 µg/mL of purified anti-CD4 IgGs from ART-treated HIV+ cocaine users (HIV+/D), anti-CD4 IgGs from ART-naive HIV+ cocaine users (Neg1), total IgGs (Neg2) from ART-treated HIV+ cocaine users and an anti-CD4 mAb (Pos). The percent of CD4+ T cytolysis was assessed using flow cytometry. Non-parametric Mann–Whitney *U* and Spearman correlation tests. *p<0.05; **p<0.01.

To investigate the effect of cocaine on HIV pathogenesis, we evaluated the plasma levels of anti-CD4 IgG and CD4+ T cell counts in HIV+ cocaine users and HIV+ non-users, as well as uninfected cocaine users, and uninfected non-users. Notably, HIV+ cocaine users had increased plasma anti-CD4 IgGs and decreased CD4+ T cell counts compared to all control groups (Figure 1A and B). Furthermore, the level of anti-CD4 IgG was inversely correlated with CD4+ T cell counts in HIV+ cocaine users but not in HIV+ non-users (Figure 1C). There was no correlation between anti-CD4 IgG and CD4+ T cell counts in uninfected subjects (data not shown). The anti-CD4 IgGs purified from HIV+ cocaine users on suppressive ART mediated ADCC. Further, the anti-CD4 IgGs purified from ART-naive HIV+ cocaine users and total IgGs derived from HIV+ cocaine users failed to induce CD4+ T cell death *in vitro* (Figure 1D).

### Chronic cocaine use in HIV+ individuals was associated with increased B cell TLR4 expression and B cell activation

**Figure 2:**
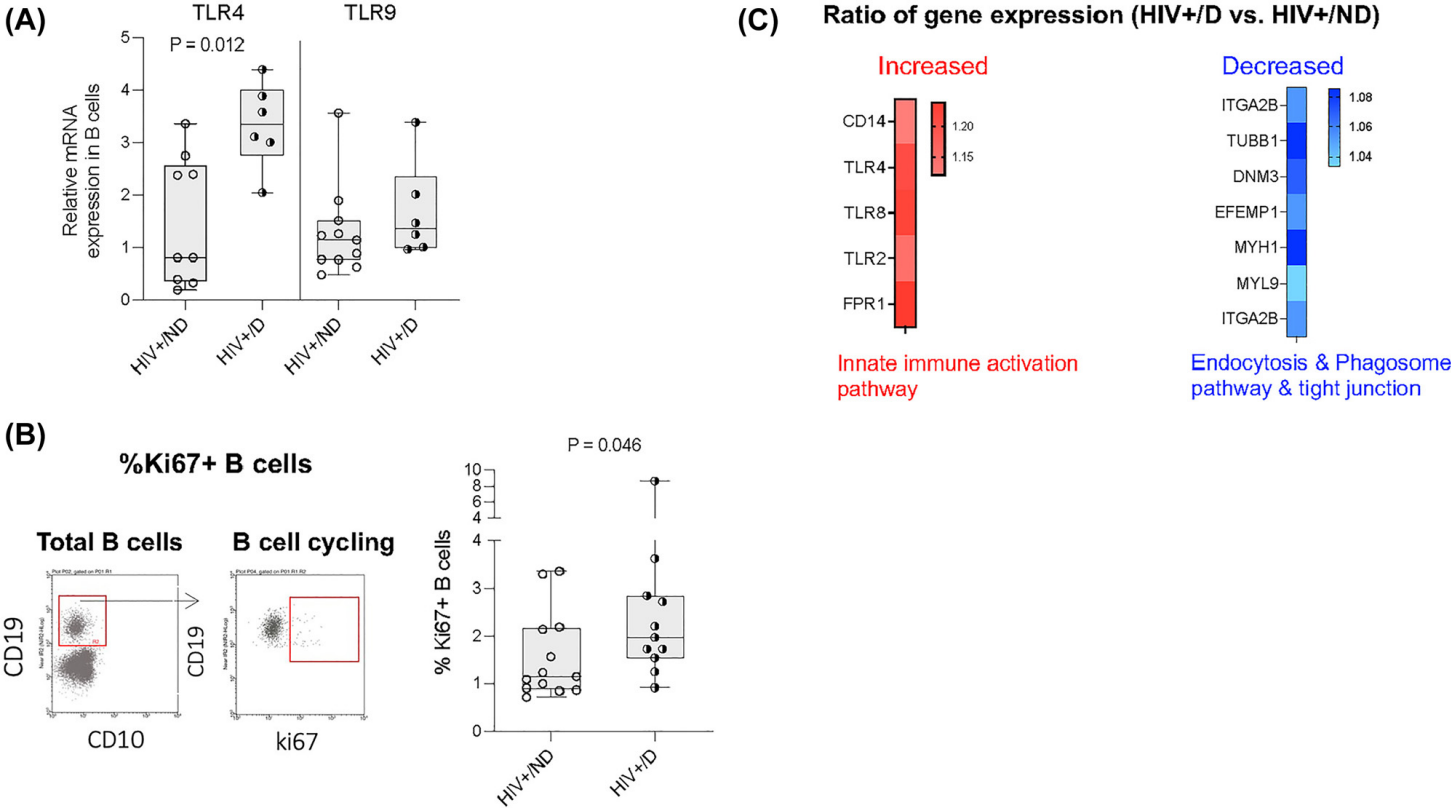
Cocaine use was associated with B cell activation and innate immune activation in HIV. B cells were isolated from PBMCs from HIV+ subjects using a negative selection kit, TLR4 and TLR9 mRNA expression was evaluated using qPCR. (A) TLR4 and TLR9 mRNA relative expression to GAPDH was shown in non-users and cocaine users with HIV. (B) The representative dot plots and summarized data on the percentages of B cell cycling expression in fresh blood samples was assessed by flow cytometry. (C) Gene expression was analyzed in isolated B cells by RNA-seq to compare between aviremic ART-treated HIV+ cocaine users (HIV+/D) and aviremic ART-treated HIV+ non-users (HIV+/ND). Expression of various gene encoding products in selected pathways showed increased (red) or decreased (blue) expression in HIV+/D compared to HIV+/ND subjects. Non-parametric Mann–Whitney *U* tests. *p<0.05.

**Figure 3:**
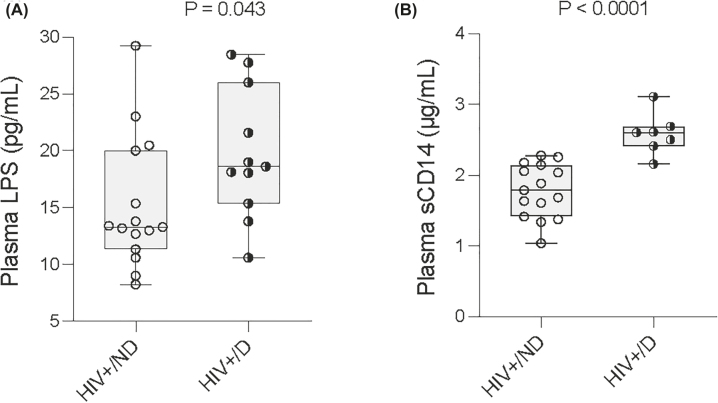
Increased plasma levels of sCD14 and LPS in HIV+ cocaine users. Plasma samples from aviremic ART-treated HIV+ cocaine users (HIV+/D) and non-users (HIV+/ND) were collected. The median plasma levels of LPS (A) and sCD14 were assessed in the two groups. Non-parametric Mann–Whitney U tests. *p<0.05; ****p<0.0001.

TLRs link to autoreactive B cell activation and autoantibody production [21]. To investigate whether increased anti-CD4 autoantibodies in HIV+ cocaine users are associated with B cell perturbations, we evaluated TLR4 and TLR9 mRNA relative expression in isolated B cells from two HIV+ study group subjects. Intriguingly, TLR4 but not TLR9 mRNA expression in B cells was increased in HIV+ cocaine users compared to HIV+ non-users (Figure 2A). Moreover, the percentages of cycling B cells, an activation marker for B cells, were increased in HIV+ cocaine users compared to HIV+ non-users (Figure 2B). We also analyzed B cell signatures between HIV+ cocaine user and HIV+ non-user groups. Intriguingly, we found genes related to the TLR signaling pathways in sensing microbial products (e.g., LPS) and innate immune activation such as TLR2, TLR4, TLR8, CD14, and FPR1 expressed higher in HIV+ cocaine users compared to HIV+ non-users (Figure 3C). On the contrary, HIV+ cocaine users showed decreased gene expression ITGA2B, TUBB1, DNM3, EFEMP1, MYH1, MYL9, and ITGA2, related to endocytosis, phagosome pathway, and tight junction (Figure 2C). These results suggest that cocaine use is associated with TLR or TLR ligand (e.g., LPS)-mediated B cell perturbations in HIV.

### Chronic cocaine use was associated with increased plasma levels of LPS and sCD14 in HIV

Next, to investigate if cocaine use alters plasma levels of TLR ligands, we evaluated the plasma levels of LPS and sCD14, markers of systemic microbial translocation [28], [29], [30]. Notably, both plasma LPS and sCD14 levels were increased in HIV+ cocaine users compared to HIV+ non-users (Figure 3A and B). These results indicate that HIV+ subjects with chronic cocaine use had increased systemic microbial translocation compared to HIV+ non-users.

## Discussion

In HIV infection, circulating CD4+ T cell counts predict disease progression regardless of ART [31]. Although the mechanism of poor CD4+ T cell recovery from ART in HIV has been extensively studied, none of these studies have proposed a mechanism that specifically targets CD4+ T cells [32]. We were the first group to report that anti-CD4 IgGs mediate CD4+ T cell death and poor immune recovery under ART [33]. Nonetheless, the link between substance use and B cell perturbations in HIV remains unknown. In this study, we show that the levels of anti-CD4 IgG antibody remain elevated in HIV+ individuals on suppressive ART. Chronic cocaine use is associated with anti-CD4 IgG production, signatures of B cell activation via innate immune activation, and poor CD4+ T cell recovery in HIV+ individuals on suppressive ART. These results suggest a causal and pathologic role of anti-CD4 IgGs in blunting CD4+ T cell recovery in HIV+ cocaine users, even with good evidence of ART adherence (e.g., aviremic).

Cocaine affects HIV progression independently of ART [5], and cocaine users consistently display inflammation and substantial mortality and morbidity [34]. Dopamine receptors are receptors for cocaine expressed mainly in neurons and also in tissues (e.g., gut) [35]. Cocaine use/abuse is associated with intestinal symptoms (e.g., constipation and vomiting) and dysfunction, as well as “leaky” gut and gut microbial dysbiosis [36]. A recent study of cocaine administration in mice resulted in a comprised gut barrier [36]. Thus, cocaine may induce gut dysfunction directly. Cocaine may also affect gut function indirectly through the perturbation of the brain-gut axis or gut microbial dysbiosis. Furthermore, cocaine induces cellular activation that may associate with TLRs [37]. We and other colleagues reported that HIV and cocaine both contribute to an impaired gut barrier integrity [36, 38]; ART partially recovers gut mucosal damage and greatly reduces microbial translocation and improves CD4+ T cell recovery [39]. However, a subgroup of HIV patients cannot restore their CD4+ T cell counts as healthy individuals; they exhibit increased inflammation, microbial translocation, mortality, and morbidity. Other drugs such as opioid use disorders may have similar effects on gut permeability and microbial translocation [40, 41], resulting in B cell perturbations, anti-CD4 IgG production, and blunted CD4+ T cell reconstitution, which deserves further investigations.

Based on the RNA-seq analysis of B cells, HIV+ cocaine users exhibited decreased gene expression related to tight junctions (Figure 2C). As a consequence, HIV+ chronic cocaine users experience persistent microbial product translocation and inflammation. Moreover, long-term repeated stimulation of microbial products (e.g., LPS) are associated with increased TLR4 expression in B cells and the breakdown of B cell tolerance in HIV+ cocaine users, which is consistent with increased TLRs signaling and innate immune activation pathway enrichment by RNA-seq analysis (Figure 2C). We also found reduced gene expression of endocytosis and phagosome pathway in HIV+ cocaine users compared to those in HIV+ non-users, indicating a decreased clearance of apoptotic debris along with the immune complex [42]. As a result, the interaction of hyperactivated B cells with HIV gp120-CD4 protein may result in pathogenic anti-CD4 IgG production, and anti-CD4 IgGs mediate CD4+ T cell death via ADCC, which prevents CD4+ T cell recovery. As previously reported, CD4+ T cell depletion contributes to increased mortality and morbidity even after long-term viral suppression on ART [43] (Graphical abstract).

HIV itself may also play a role in anti-CD4 pathogenic autoantibody production. HIV Tat and gp120 proteins directly reduce tight junction expression in epithelial cells *in vitro;* decreased tight junction protein expression was found in gut tissues from patients under viral-suppressive ART *ex vivo*. Pathogenic autoantibodies are produced post-ART but not pre-ART, and autoimmune diseases in HIV often develop after ART [44]. Even under viral-suppressive ART, HIV still can actively replicate in the B cell follicles of lymph nodes (probably due to the lack of CD8^+^ T cell cytotoxicity and lack of access to treatment) and/or tissues in some HIV-infected individuals [45]. Therefore, CD4 protein released from remaining higher rate of apoptotic CD4+ T cells in some HIV-infected individuals on suppressive ART [46] interact with HIV gp120 in the lymph nodes, and may promote anti-CD4 autoantibody production in response to gp120-CD4 complex in the presence of elevated residual inflammation, increased TLR4 and TLR4 ligands (e.g., LPS), and modest B cell activation (producing high-affinity antibodies) but not excessive B cell activation (producing low-affinity antibodies) [47]. Indeed, increased plasma levels of microbial TLR-downstream proinflammatory cytokines are associated with cocaine use (e.g., IL-6, sCD14, and Figure 3) [34]. TLR signals in B cells play a role in autoantibody production and autoimmune disease [21]. Thus, long-term repeated stimulation of bacterial products may induce breakdown of B cell tolerance as shown in previous studies that altered BCR and microbial TLR signals (e.g., TLR4 and MyD88) may promote the breakdown of autoimmunity [23]. However, it is not fully understood how microenvironmental and inflammatory factors drive the breakdown of B cell tolerance and produce pathogenic autoantibodies, especially in humans.

In a longitudinal study [48], HIV+ non-drug users demonstrated to have the successful CD4+ T cell reconstitution, but HIV+ drug users (e.g., cannabis, cocaine, opioid) failed to reconstitute their CD4+ T cells following two years of ART, even with similar pre-ART CD4+ T cell counts. Anti-CD4 IgGs purified from ART-naive HIV+ cocaine users failed to mediate ADCC against CD4+ T cells (Figure 2D). This result is consistent with previous studies which showed that ART-naive HIV+ individuals had B cell polyclonal activation including increased plasma anti-CD4 lgG levels, which purified anti-CD4 IgGs from plasma did not mediate ADCC activity [49]. The non-functional anti-CD4 IgGs in untreated patients likely are induced by a viremia-mediated cytokine storm, because inflammation can induce non-functional autoantibodies non-specifically, as shown in alcoholic liver disease [50]. In summary, anti-CD4 IgGs from HIV+ cocaine users have ADCC activity and may play a role in poor CD4+ T cell recovery from suppressive ART.

One limitation of the current study is the small sample size. Nonetheless, this is the first report on B cell perturbations and cocaine use in ART outcomes in HIV. The lack of understanding of poor immune reconstitution from ART in HIV+ cocaine users represents a critical barrier to our understanding of drug-accelerated HIV pathogenesis and significantly hinders our ability to treat patients who fail to restore CD4+ T cell counts under traditional ART. Therefore, it is critical to develop new treatments to improve CD4+ T cell recovery and reduce mortality and morbidity for HIV+ drug users in addition to ART.

## References

[1] Grossman Z, Meier-Schellersheim M, Paul WE, Picker LJ (2006). Pathogenesis of HIV infection: what the virus spares is as important as what it destroys. Nat Med.

[2] Okoye AA, Picker LJ (2013). CD4(+) T-cell depletion in HIV infection: mechanisms of immunological failure. Immunol Rev.

[3] Luo ZWLZ, Martin L, Wan Z, Meissner EG, Espinosa E, Wu H (2017). Pathological role of anti-CD4 antibodies in HIV-infected immunologic non-responders under viral suppressive antiretroviral therapy. J Infect Dis.

[4] Strathdee SA, Stockman JK (2010). Epidemiology of HIV among injecting and non-injecting drug users: current trends and implications for interventions. Curr HIV AIDS Rep.

[5] Baum MKRC, Lai S, Sales S, Page B, Campa A (2009). Crack-cocaine use accelerates HIV disease progression in a cohort of HIV-positive drug users. J Acquir Immune Defic Syndr.

[6] Doke M, Jeganathan V, McLaughlin JP, Samikkannu T (2021). HIV-1 Tat and cocaine impact mitochondrial epigenetics: effects on DNA methylation. Epigenetics.

[7] Shu C, Justice AC, Zhang X, Wang Z, Hancock DB, Johnson EO (2020). DNA methylation mediates the effect of cocaine use on HIV severity. Clin Epigenet.

[8] Dhillon NK, Peng F, Bokhari S, Callen S, Shin SH, Zhu X (2008). Cocaine-mediated alteration in tight junction protein expression and modulation of CCL2/CCR2 axis across the blood-brain barrier: implications for HIV-dementia. J Neuroimmune Pharmacol.

[9] Zenon F, Segarra AC, Gonzalez M, Melendez LM (2014). Cocaine potentiates cathepsin B secretion and neuronal apoptosis from HIV-infected macrophages. J Neuroimmune Pharmacol.

[10] Aksenova M, Sybrandt J, Cui B, Sikirzhytski V, Ji H, Odhiambo D (2020). Inhibition of the dead box RNA helicase 3 prevents HIV-1 Tat and cocaine-induced neurotoxicity by targeting microglia activation. J Neuroimmune Pharmacol.

[11] Sivalingam K, Cirino TJ, McLaughlin JP, Samikkannu T (2021). HIV-tat and cocaine impact brain energy metabolism: redox modification and mitochondrial biogenesis influence NRF transcription-mediated neurodegeneration. Mol Neurobiol.

[12] Fox HC, D’Sa C, Kimmerling A, Siedlarz KM, Tuit KL, Stowe R (2012). Immune system inflammation in cocaine dependent individuals: implications for medications development. Hum Psychopharmacol.

[13] Moreira FP, Medeiros JR, Lhullier AC, Souza LD, Jansen K, Portela LV (2016). Cocaine abuse and effects in the serum levels of cytokines IL-6 and IL-10. Drug Alcohol Depend.

[14] Mantri CK, Pandhare Dash J, Mantri JV, Dash CC (2012). Cocaine enhances HIV-1 replication in CD4+ T cells by down-regulating MiR-125b. PLoS One.

[15] Dhillon NK, Williams R, Peng F, Tsai YJ, Dhillon S, Nicolay B (2007). Cocaine-mediated enhancement of virus replication in macrophages: implications for human immunodeficiency virus-associated dementia. J Neurovirol.

[16] Volpe GE, Ward H, Mwamburi M, Dinh D, Bhalchandra S, Wanke C (2014). Associations of cocaine use and HIV infection with the intestinal microbiota, microbial translocation, and inflammation. J Stud Alcohol Drugs.

[17] Castro FOF, Silva JM, Dorneles GP, Barros JBS, Ribeiro CB, Noronha I (2019). Distinct inflammatory profiles in HIV-infected individuals under antiretroviral therapy using cannabis, cocaine or cannabis plus cocaine. AIDS.

[18] Guiducci C, Gong M, Xu Z, Gill M, Chaussabel D, Meeker T (2010). TLR recognition of self nucleic acids hampers glucocorticoid activity in lupus. Nature.

[19] Hornung V, Rothenfusser S, Britsch S, Krug A, Jahrsdorfer B, Giese T (2002). Quantitative expression of toll-like receptor 1-10 mRNA in cellular subsets of human peripheral blood mononuclear cells and sensitivity to CpG oligodeoxynucleotides. J Immunol.

[20] Pisitkun P, Deane JA, Difilippantonio MJ, Tarasenko T, Satterthwaite AB, Bolland S (2006). Autoreactive B cell responses to RNA-related antigens due to TLR7 gene duplication. Science.

[21] Lartigue A, Colliou N, Calbo S, Francois A, Jacquot S, Arnoult C (2009). Critical role of TLR2 and TLR4 in autoantibody production and glomerulonephritis in lpr mutation-induced mouse lupus. J Immunol.

[22] Simchoni N, Cunningham-Rundles C (2015). TLR7- and TLR9-responsive human B cells share phenotypic and genetic characteristics. J Immunol.

[23] Kolhatkar NS, Brahmandam A, Thouvenel CD, Becker-Herman S, Jacobs HM, Schwartz MA (2015). Altered BCR and TLR signals promote enhanced positive selection of autoreactive transitional B cells in Wiskott-Aldrich syndrome. J Exp Med.

[24] Sindberg GM, Sharma U, Banerjee S, Anand V, Dutta R, Gu CJ (2015). An infectious murine model for studying the systemic effects of opioids on early HIV pathogenesis in the gut. J Neuroimmune Pharmacol.

[25] Fu X, Cheng D, Luo Z, Wagner A, Fitting S, Cong X (2022). Oral enrichment of Streptococcus and its role in systemic inflammation related to monocyte activation in humans with cocaine use disorder. J Neuroimmune Pharmacol.

[26] Smyth GK, Speed T (2003). Normalization of cDNA microarray data. Methods.

[27] Rothman KJ (1990). No adjustments are needed for multiple comparisons. Epidemiology.

[28] Jiang W, Younes SA, Funderburg NT, Mudd JC, Espinosa E, Davenport MP (2014). Cycling memory CD4+ T cells in HIV disease have a diverse T cell receptor repertoire and a phenotype consistent with bystander activation. J Virol.

[29] Lyons JL, Uno H, Ancuta P, Kamat A, Moore DJ, Singer EJ (2011). Plasma sCD14 is a biomarker associated with impaired neurocognitive test performance in attention and learning domains in HIV infection. J Acquir Immune Defic Syndr.

[30] Sandler NG, Wand H, Roque A, Law M, Nason MC, Nixon DE (2011). Plasma levels of soluble CD14 independently predict mortality in HIV infection. J Infect Dis.

[31] Pasternak AODBM, Bakker M, Berkhout B, Prins JM (2015). High current CD4+ T cell count predicts suboptimal adherence to antiretroviral therapy. PLoS One.

[32] Gaardbo JC, Hartling HJ, Gerstoft J, Nielsen SD (2012). Incomplete immune recovery in HIV infection: mechanisms, relevance for clinical care, and possible solutions. Clin Dev Immunol.

[33] Luo Z, Li Z, Martin L, Wan Z, Meissner EG, Espinosa E (2017). Pathological role of anti-CD4 antibodies in HIV-infected immunologic nonresponders receiving virus-suppressive antiretroviral therapy. J Infect Dis.

[34] Rahimian P, He JJ (2016). HIV/neuroAIDS biomarkers. Prog Neurobiol.

[35] Li ZS, Schmauss C, Cuenca A, Ratcliffe E, Gershon MD (2006). Physiological modulation of intestinal motility by enteric dopaminergic neurons and the D2 receptor: analysis of dopamine receptor expression, location, development, and function in wild-type and knock-out mice. J Neurosci.

[36] Chivero ET, Ahmad R, Thangaraj A, Periyasamy P, Kumar B, Kroeger E (2019). Cocaine induces inflammatory gut milieu by compromising the mucosal barrier integrity and altering the gut microbiota colonization. Sci Rep.

[37] Liao K, Guo M, Niu F, Yang L, Callen SE, Buch S (2016). Cocaine-mediated induction of microglial activation involves the ER stress-TLR2 axis. J Neuroinflammation.

[38] Brenchley JM, Price DA, Schacker TW, Asher TE, Silvestri G, Rao S (2006). Microbial translocation is a cause of systemic immune activation in chronic HIV infection. Nat Med.

[39] Jiang W, Lederman MM, Hunt P, Sieg SF, Haley K, Rodriguez B (2009). Plasma levels of bacterial DNA correlate with immune activation and the magnitude of immune restoration in persons with antiretroviral-treated HIV infection. J Infect Dis.

[40] Meng J, Banerjee S, Zhang L, Sindberg G, Moidunny S, Li B (2019). Opioids impair intestinal epithelial repair in HIV-infected humanized mice. Front Immunol.

[41] Hileman CO, Bowman ER, Gabriel J, Kettelhut A, Labbato D, Smith C (2022). Impact of heroin and HIV on gut integrity and immune activation. J Acquir Immune Defic Syndr.

[42] Koppensteiner H, Brack-Werner R, Schindler M (2012). Macrophages and their relevance in human immunodeficiency virus type I infection. Retrovirology.

[43] Lapadula G, Cozzi-Lepri A, Marchetti G, Antinori A, Chiodera A, Nicastri E (2013). Risk of clinical progression among patients with immunological nonresponse despite virological suppression after combination antiretroviral treatment. AIDS.

[44] Iordache L, Launay O, Bouchaud O, Jeantils V, Goujard C, Boue F (2014). Autoimmune diseases in HIV-infected patients: 52 cases and literature review. Autoimmun Rev.

[45] Rothenberger MK, Keele BF, Wietgrefe SW, Fletcher CV, Beilman GJ, Chipman JG (2015). Large number of rebounding/founder HIV variants emerge from multifocal infection in lymphatic tissues after treatment interruption. Proc Natl Acad Sci U S A.

[46] Luo Z, Zhou Z, Ogunrinde E, Zhang T, Li Z, Martin L (2017). The effect of plasma auto-IgGs on CD4+ T cell apoptosis and recovery in HIV-infected patients under antiretroviral therapy. J Leukoc Biol.

[47] Luo Z, Ma L, Zhang L, Martin L, Wan Z, Warth S (2016). Key differences in B cell activation patterns and immune correlates among treated HIV-infected patients versus healthy controls following influenza vaccination. Vaccine.

[48] Jiang W, Luo Z, Martin L, Wan Z, Fu P, Wagner A (2018). Drug use is associated with anti-CD4 IgG-mediated CD4+ T cell death and poor CD4+ T cell recovery in viral-suppressive HIV-infected individuals under antiretroviral therapy. Curr HIV Res.

[49] Chams V, Idziorek T, Klatzmann D (1991). Biological properties of anti-CD4 autoantibodies purified from HIV-infected patients. AIDS.

[50] Lian M, Hua J, Sheng L, Qiu DK (2013). Prevalence and significance of autoantibodies in patients with alcoholic liver disease. J Dig Dis.

